# Social and cultural factors underlying generational differences in overweight: a cross-sectional study among ethnic minorities in the Netherlands

**DOI:** 10.1186/1471-2458-11-105

**Published:** 2011-02-16

**Authors:** Karen Hosper, Mary Nicolaou, Irene van Valkengoed, Vera Nierkens, Karien Stronks

**Affiliations:** 1 Department of Public Health, Academic Medical Centre - University of Amsterdam, Meibergdreef 15, 1105 AZ, Amsterdam, the Netherlands; 2 PHAROS, Knowledge and Advisory Centre on Migrants, Refugees and Health, Herenstraat 35, 3507 LH Utrecht, the Netherlands

## Abstract

**Background:**

The prevalence of overweight appears to vary in people of first and second generation ethnic minority groups. Insight into the factors that underlie these weight differences might help in understanding the health transition that is taking place across generations following migration. We studied the role of social and cultural factors associated with generational differences in overweight among young Turkish and Moroccan men and women in the Netherlands.

**Methods:**

Cross-sectional data were derived from the LASER-study in which information on health-related behaviour and socio-demographic factors, level of education, occupational status, acculturation (cultural orientation and social contacts), religious and migration-related factors was gathered among Turkish and Moroccan men (n = 334) and women (n = 339) aged 15-30 years. Participants were interviewed during a home visit. Overweight was defined as a Body Mass Index ≥ 25 kg/m^2^. Using logistic regression analyses, we tested whether the measured social and cultural factors could explain differences in overweight between first and second generation ethnic groups.

**Results:**

Second generation women were less often overweight than first generation women (21.8% and 45.0% respectively), but this association was no longer significant when adjusting for the socioeconomic position (i.e. higher level of education) of second generation women (Odds Ratio (OR) = 0.77, 95%, Confidence Interval (CI) 0.40-1.46). In men, we observed a reversed pattern: second generation men were more often overweight than first generation men (32.7% and 27.8%). This association (OR = 1.89, 95% CI 1.09-3.24) could not be explained by the social and cultural factors because none of these factors were associated with overweight among men.

**Conclusions:**

The higher socio-economic position of second generation Turkish and Moroccan women may partly account for the lower prevalence of overweight in this group compared to first generation women. Further research is necessary to elucidate whether any postulated socio-biological or other processes are relevant to the opposite pattern of overweight among men.

## Background

Higher rates of overweight and obesity have been observed among ethnic minority groups compared to host populations [1-4]. In addition, there appears to be a large variation in overweight according to generational status in these groups. Several studies show a convergence of overweight across generations towards the level found in the host population [1-3,5]. In line with these findings, in the Netherlands the likelihood of being overweight was lower among young (aged 15-30 years) Turkish and Moroccan women born in the Netherlands (second generation), than in first generation young women born in Turkey or Morocco [6]. This implies a convergence towards the prevalence rates among Dutch young women. However, we found the opposite to be true in first and second generation young Turkish and Moroccan men within this age group. Prevalence of overweight among second generation men tend to be higher in the second generation compared to the first generation. We found no exactly similar studies, however, a pattern of second generation migrant women being less centrally obese than first generation women with the opposite among men, has been seen before [7]. Most other studies did not compare generations with this young age group which makes it difficult to compare the results [1,3,8].

The generational differences in body weight could reflect differences in weight-related behaviours in people of first and second generations. Prior studies have shown, for example, that second generation ethnic minority groups are more physically active compared to the first generation, which may prevent the development of overweight within these groups [9,10]. Yet, very little is known about the underlying social and cultural processes that lead to a different weight status across generations of ethnic minorities and why this pattern may differ for men and women. Insight into these processes would help us to better understand the transition in health that is taking place over generations. This information could be useful for forecasting trends in the prevalence of overweight and associated diseases within ethnic minority groups. In addition, when the underlying cause of the changes in overweight prevalence is known, health promotion can be directed more effectively towards the high risk groups within ethnic minority populations.

Two important processes that have been associated with differences of overweight in people from ethnic minorities are the changes that have taken place in their socioeconomic position and the process of acculturation they have gone through. On the first point, a better socioeconomic position in terms of education and occupation has been associated with a lower prevalence of overweight in several populations, including Turks and Moroccans [4,11,12]. Although the social patterning for men and women has become more similar as a result of globalization, women still show more consistent associations between socioeconomic position and overweight compared with men [12]. Therefore, socioeconomic position may play a different role for men and women in the development of overweight across generations. As far as second generation women are concerned, their higher socioeconomic position (more highly educated and higher position on the labour market) may be related to more Western norms and values with regard to body weight and lifestyle and therefore account for the lower rates of overweight found in this group [13].

Secondly, besides socioeconomic differences, the second generation groups are generally more socially and culturally integrated in the host culture, when compared to the first generation groups [14]. This so-called process of acculturation has been associated with overweight in several ethnic minority populations [15,16]. Acculturation is often defined as 'a process of cultural changes as a result of contact with the dominant culture'[17]. Greater preference for English language use, which is a commonly used indicator for acculturation in the USA, has been associated with a lower prevalence of overweight in groups that already have high rates of overweight - such as Mexican Americans [18]. In turn, English language use is associated with more overweight in populations who were relatively less often overweight at the time they migrated to the USA, such as Asian Americans and Puerto Ricans [19]. With regard to the gender differences across two generations of young Turkish and Moroccan groups, we expect that the impact of acculturation on overweight may differ between the groups. There are indications that non-western women of the second generation who acculturate tend to take over a wish to be thin while men take over unhealthy habits such as eating snacks and fast-food and are less concerned about their body image [13].

Besides socioeconomic and acculturation factors, socio-demographic (e.g. marital status, having children), migration-related or religious factors also differ in general between generations and may be possible underlying factors for generational differences in overweight [11]. For example, first generation Turkish and Moroccan people often marry (and have children) at a younger age which in turn is associated with a greater risk for developing overweight [14]. The region where these people originally came from, rural or urban for example, might also influence their lifestyle [20]. With regard to religion, the stronger attachment to religion in first generation groups could possibly create barriers for physical activity behaviour and consequently result in more overweight [21]. On this point, women may be more affected than men as participation in sports is less common in women from non-western cultures.

In a previous study, we demonstrated differences in overweight in first and second generation Turkish and Moroccan men and women in the Netherlands [6]. The objective of the present study is to assess which social and cultural factors might account for these generational differences in overweight and how they may differ for men and women.

## Methods

We analyzed data collected in 2003-2004 in the LASER study (Lifestyle in Amsterdam: a Study among Ethnic gRoups). The aim of this study was to gain insight into health-related risk factors and its determinants in young Turkish and Moroccan people living in Amsterdam, the Netherlands. For this study, a random sample was drawn from the Amsterdam population registry, which included people between the ages of 10 and 30 years who had either been born in Turkey or Morocco (first generation) or in the Netherlands with at least one parent who had been born in Turkey or Morocco (second generation). During a home visit, trained interviewers with a similar ethnic background and sex to the participant, conducted face-to-face interviews using a structured questionnaire. This questionnaire was then forward-translated and back-translated into Turkish and Moroccan-Arabic by professional translators.

For the current study, we included Turkish and Moroccan participants aged 15 to 30 years. The main reason for excluding the 10-14 years olds is that 90% of this group belongs to the second generation, which would lead to less reliable assessment of generational differences. In addition, we assume that the influence of social and cultural determinants on overweight may be different within this younger age group.

Of the Turkish sample (aged 15-30) in total 997 persons were contacted at their home address. Of these persons 33% refused to participate in the study and 14% of these persons could not be reached after three attempts of visiting their home. In total 52% of the Turkish sample participated in the study, which resulted in 519 participants. Of the Moroccan sample 601 persons were contacted at their home address. Of these persons 30% refused to participate in the study and 23% was not reached after three attempts. In total 46% of the Moroccan sample participated in the study which resulted in 277 participants. Due to many missing or invalid data (15%) on weight and height, the total numbers of participants used in our analyses were 249 Moroccan and 424 Turkish men and women.

The study sample appeared to be representative of the Turkish and Moroccan populations (aged 15-30 years) living in Amsterdam and according to sex, generational status, city district and educational level. With regard to age, among the Moroccan male sample, the 20-30 year-olds were slightly under represented compared to the general population of Moroccans in Amsterdam.

We certify that all applicable institutional and governmental regulations concerning the ethical use of human volunteers were followed during this research. The study was approved by the Medical Ethical Committee of the Academic Medical Centre in Amsterdam.

### Overweight

Body mass index (BMI) was calculated as weight (kg) divided by height (m^2^). For people of 18 years and older, overweight was defined as a BMI of 25 or higher. For people of 15 up to 17 years, we used the recommended sex and age-adjusted cut-off points [22]. The weight and height of the participants were measured during the home visits. Weight was measured on an electronic scale to the nearest 0.1 kg after removal of shoes, jackets, heavier clothing and pocket contents. Height was measured twice, standing in an upright position without shoes, with a measuring tape to the nearest 0.1 cm. Due to some logistical problems, not all participants were measured during the home visit. In these cases, which were completely random, weight and height were self-reported (43% of the cases, n = 289). Chi-square tests showed that the self-reported and the measured group did not differ on any of the dependent and independent variables used in this study. The consequences of this measurement issue for the results of the study are discussed in the limitations section (discussion) of this paper.

### Socio-demographic factors

Age (years) and ethnicity (Turkish, Moroccan) were treated as confounders in the relationship between generational status (first or second generation) and overweight (yes, no), and the potential determinants. Marital status (married or cohabiting, not married/not cohabiting) and having children (yes, no) were considered as potential explanatory factors in the association between overweight and generational status.

### Socioeconomic position

Firstly, *Educational level *was indicated by the highest level of education attained for those people who had finished school. For those people who were still following a course of study, the current level of education was used. Educational level was categorized as "low" when people had no education or only a primary school education and "moderate" when people had had lower to intermediate level vocational training. Participants were considered to have a "high" level of education when they had completed higher professional education or university.

To justify this categorisation for students as well as non-students, we would like to refer to the fact that, unlike other European countries (such as the UK), the educational system in the Netherlands is characterized by the process of streaming. This implies that pupils from the age of 12 years start an educational program at a certain level. This starting position is a good predictor for the level of education they will finally achieve and is also associated with their future position on the labour market [23].

Secondly, *position on the labour market *was measured by the current daily 'main activity' of the participants. We divided the participants into four categories: 1) unemployed, 2) homemakers, 3) paid employment, and 4) students still studying. Among men, there was only one homemaker, therefore this category was left out within the logistic regression analyses in men.

Thirdly, we measured *occupational status *in which persons were categorized according to the highest occupational status within the family. For adolescents who were still living with their parents, we used the highest occupational position of the father or mother. In cases where one or both parents were retired, we used their level of occupation prior to retirement. For young adults with their own household, we used the highest occupation of the participant and his/her partner. The following categories were distinguished, using a standard classification of occupations [24]: 1) manual occupation (i.e. cleaning jobs), 2) non-manual occupation (i.e. administrative work), 3) unemployed, and 4) students.

### Migration related factors

*Region of origin *was measured by asking participants if they (themselves or their family) originally came from a small village/town or from a big city in their country of origin.

In addition, participants were asked what had been the *main reason for migration *to the Netherlands. First generation participants answered this question for themselves and second generation participants were asked to indicate the main reason for migration of their parents. The reasons were categorized as follows: 1) came with parents, 2) family reunion/marriage or 3) economic reasons, such as education or employment.

### Acculturation

The indicators of acculturation were based on Berry's approach whereby this position is considered in terms of orientation towards the majority culture versus culture of origin, and social contacts with the host population versus contacts with people from the culture of origin [25]. Thus the following components were derived.

Firstly, *cultural orientation *was measured by 10 items about language use with family members and friends, use of media, difficulties with reading Dutch, shopping preferences and emancipation as an example of Western norms and values [26,27]. For example, one item on the use of media was as follows: "How often do you watch Dutch television programmes?", but also, "How often do you watch Turkish television programmes?" This enabled participants to answer positive to both items which would indicate their bi-cultural orientation. The cultural orientation scale was constructed using principal component analysis and reliability analysis (alpha = .64). Secondly, *social contacts *were measured by three questions about contacts with native Dutch people during leisure time (i.e., How many of your best friends are ethnic Dutch?) (alpha = .84). For both scales, the scores on the items in each scale were summed up. A mean substitution was made for cases where one item was missing. In total there were 7 male participants and 6 female participants with missing items on one of both acculturation scales.

The final scales were categorized in tertiles in order to denote an individual's cultural position, with subjects in the first tertile being the least oriented towards the majority culture (having the least contacts with ethnic Dutch) and those in the third tertile being the most oriented towards the majority culture (having the most contacts with ethnic Dutch).

### Religion

In addition, we measured the perceived *importance of religion *which was scored on a 4-point scale ranging from not important at all (1) to very important (4), which was dichotomized into very important (score 4) versus not, to moderately important (score 1-3).

### Analyses

First we performed all analyses separately for Turks and Moroccans to check that the pattern of associations was similar by ethnicity. It appeared that all associations between social and cultural factors and overweight were in the same direction in both groups. Also, the associations between generational status and overweight were similar in both groups and for women and men. Subsequently, we decided to combine the Turkish and Moroccan groups as ethnicity would not affect the associations.

To assess the extent to which the potential determinants differed between the generations, we calculated percentages by gender and generational status using cross tabulations with Chi-square tests. To investigate which factors could account for the generational differences in overweight, we conducted logistic regression analyses. First we assessed the generational differences in a crude model (adjusted for age and ethnicity). Secondly, we added the separate social and cultural factors to the crude model with generational status as an independent variable and overweight as a dependent variable. In Model 1 we added socio-demographic factors, in Model 2 we added socioeconomic position, in Model 3 we added migration-related factors and in Model 4 acculturation and religion were added to the crude model. All logistic regression analyses were adjusted for age and ethnicity. The results are presented as Odds Ratios (OR) with 95% Confidence Intervals (CI).

## Results

### Study characteristics

Table 1 shows the characteristics of the study population by gender and generational status. First generation ethnic groups were generally older, more often married and more frequently had children compared to the second generation. Among first generation women, more Turkish than Moroccan women were represented. Among men there were no differences between the generations with regard to ethnic background. Approximately two-thirds of the first generation group had migrated to the Netherlands after the age of 6 years. In women, we found more significant generational differences in socioeconomic position than in men, with second generation women being higher educated than first generation women. Among women, the first generation had migrated more often for reasons of marriage or family reunions, while (the family of) the second generation had mostly migrated for economic reasons (education or employment). No generational differences were found in men for the reason of migration. For both men and women the cultural and religious factors generally differed between the generations with lower cultural orientation, fewer social contacts and religion being more important among first generation migrants. The associations with cultural and religious factors were slightly stronger in women than in men.

**Table 1:** Study characteristics by gender and generational status

	Turkish and Moroccan men N = 334			Turkish and Moroccan women N = 339		
	1st generation N= 126 % (n)	2nd generation N= 208 % (n)	p-value men	1st generation N= 160 % (n)	2nd generation N= 179 % (n)	p-value women
Overweight or obese BMI (kg/m2) ≥25	27.8 (35)	32.7 (68)	.210	45.0 (72)	21.8 (39)	.000
**Socio-demographic factors**						
*Age*			.000			.000
15-19	39.7 (50)	60.1 (125)		21.9 (35)	60.3 (108)	
20-24	22.2 (28)	25.5 (53)		16.9 (27)	23.5 (42)	
25-30	38.1 (48)	14.4 (30)		61.3 (98)	16.2 (29)	
*Ethnicity*			.225			.031
Turkish	71.4 (90)	66.8 (139)		63.1 (101)	52.5 (94)	
Moroccan	28.6 (36)	33.2 (69)		36.9 (59)	47.5 (85)	
Age at migration of 1st generation*						
> 6^th ^year	63.9 (78)	-		67.1 (106)	-	
< 6th year	36.1 (44)	-		32.9 (52)	-	
Years of residence- 1st generation*						
< 12 years	46.8 (58)	-		42.5 (68)	-	
> 12 years	53.2 (66)	-		57.5 (92)	-	
*Marital status (married or cohabiting))*	35.7 (45)	15.9 (33)	.000	60.0 (96)	22.3 (40)	.000
*Having children living at home (yes)*	26.2 (3)	12.5 (26)	.001	56.3 (90)	19.6 (35)	.000
Socio-economic factors*						
*Educational level*			.281			.000
Low	11.7 (14)	6.9 (14)		28.4 (44)	3.4 (6)	
Moderate	58.3 (70)	64.9 (131)		50.3 (78)	67.6 (119)	
High	30.0 (36)	28.2 (57)		21.3 (33)	29.0 (51)	
*Position on labour market (individual)*			.002			.000
Unemployed	10.4 (12)	6.0 (12)		12.7 (20)	7.3 (13)	
Homemaker	-	-		35.7 (56)	7.9 (14)	
Employed	44.3 (51)	28.4 (57)		24.2 (38)	18.6 (33)	
Students	45.2 (52)	65.7 (132)		27.4 (43)	66.1 (117)	
*Occupational status (family)*			.052			.433
Unemployed	26.2 (28)	37.2 (64)		28.1 (39)	35.6 (57)	
Manual occupation	47.7 (51)	37..8 (65)		38.8 (54)	37.5 (60)	
Non-manual occupation	22.4 (24)	16.3 (28)		29.5 (41)	25.0 (40)	
Students	3.7 (4)	8.7 (15)		3.6 (5)	1.9 (3)	
Migration related factors*						
*Region of origin*			.138			.116
Big city	41.3 (50)	34.6 (71)		35.8 (57)	57.1 (100)	
Small city/village	58.7 (71)	65.4 (134)		64.2 (102)	42.9 (75)	
*Reason for migration*			.619			.000
Came with parents	8.1 (10)	10.7 (22)		11.3 (18)	13.6 (24)	
Family reunion/marriage	13.7 (17)	11.2 (23)		37.5 (60)	15.8 (28)	
For economic reasons	78.2 (97)	78.0 (160)		51.3 (82)	70.6 (125)	
Acculturation*						
*Cultural orientation*			.042			.016
1^st ^tertile: least oriented towards Dutch culture	37.6 (47)	22.7 (46)		56.8 (88)	14.1 (25)	
2^nd ^tertile	31.2 (39)	38.4 (78)		24.5 (38)	38.4 (68)	
3^rd ^tertile: most oriented towards Dutch culture	31.2 (39)	38.9 (79)		18.1 (29)	47.5 (84)	
*Social contacts*			.014			.000
1^st ^tertile:least contacts with ethnic Dutch	48.0 (60)	34.3 (70)		49.4 (79)	36.5 (65)	
2^nd ^tertile	30.4 (38)	35.8 (73)		31.9 (51)	32.6 (58)	
3^rd ^tertile: most contacts with ethnic Dutch	21.6 (27)	29.9 (61)		18.8 (30)	30.9 (55)	
**Religion **very important*	68.0 (83)	56.1 (110)	.022	72.9 (113)	70.5 (124)	.356

* Numbers do not exactly add to total number of participants due to missing cases on these variables.

### Factors associated with overweight

Marital status and having children were associated with overweight in women, but not in men (Table 2). Unmarried women were less often overweight than married women. Women without any children showed a trend of having more overweight. Regarding the socioeconomic factors, these were found to be only associated with overweight in women. Women with a moderate to high level of education were less often overweight than those with a low level of education. Women who were classed as students were less often overweight than those who were unemployed or homemakers. Among men, ethnicity was the only factor that was significantly associated with overweight - with Turkish men more often being overweight than Moroccan men. The factors relating both to migration and culture and religion were not significantly associated with overweight in women or men.

**Table 2:** The association of potential determinants with the risk of being overweight among Turkish and Moroccan men and women adjusted for age and ethnicity (Odds Ratio's with 95% confidence Intervals).

	Men N= 334 OR (95%CI)	Women N = 339 OR (95%CI)
Potential determinants of overweight		
**Socio-demographic factors**		
*Age*		
25-30	1.00	1.00
20-24	0.64 (0.33-1.21)	0.45 (0.24-0.83)
15-19	0.31 (0.17-0.55)	0.24 (0.14-0.41)
*Ethnicity*		
Moroccan	1.00	1.00
Turkish	2.02 (1.14-3.59)	1.03 (0.63-1.67)
*Marital status*		
Married or cohabiting	1.00	1.00
Not married or cohabiting	0.90 (0.43-1.91)	0.19 (0.09-0.39)
*Children*		
Children (living at home)	1.00	1.00
No children (living at home)	0.90 (0.45-1.79)	0.54 (0.29-1.00)
**Socioeconomic position**		
*Educational level*		
Low	1.00	1.00
Moderate	1.25 (0.52-3.01)	0.29 (0.14-0.60)
High	0.95 (0.37-2.44)	0.16 (0.07-0.37)
*Position on labour market (individual)*		
Unemployed	1.00	1.00
Homemaker	-	1.47 (0.63-3.44)
Employed	0.97 (0.39-2.44)	0.59 (0.25-1.37)
Students	0.61 (0.22-1.72)	0.23 (0.08-0.63)
*Occupational status (family)*		
Unemployed	1.00	1.00
Manual occupation	0.95 (0.49-1.84)	1.01 (0.53-1.92)
Non-manual occupation	1.25 (0.57-2.74)	1.03 (0.51-2.04)
Students	1.16 (0.36-3.76)	1.35 (0.25-7.35)
**Migration related factors**		
*Region of origin*		
big city	1.00	1.00
small city/village	1.37 (0.78-2.39)	1.28 (0.78-2.10)
*Reason for migration*		
came with parents	1.00	1.00
family reunion/marriage	0.81 (0.27-2.44)	1.43 (0.61-3.36)
for economic reasons (work/education)	1.24 (0.52-2.99)	1.00 (0.46-2.19)
**Acculturation**		
*Cultural orientation*		
1^st ^tertile: least oriented towards Dutch culture	1.00	1.00
2^nd ^tertile	0.87 (0.46-1.62)	0.67 (0.37-1.23)
3^rd ^tertile: most oriented towards Dutch culture	0.99 (0.54-1.81)	0.86 (0.46-1.59)
*Social contacts*		
1^st ^tertile:least contacts with ethnic Dutch	1.00	1.00
2^nd ^tertile	1.02 (0.57-1.83)	0.58 (0.33-1.03)
3^rd ^tertile: most contacts with ethnic Dutch	1.13 (0.62-2.07)	0.80 (0.44-1.48)
**Religion**: very important	1.00	1.00
normally/less important	1.18 (0.71-1.95)	0.71 (0.41-1.24)

### Factors accounting for generational differences

The crude model (adjusted for age and ethnicity) shown in Table 3 shows a significant association between generational status and overweight for both men and women. Second generation women were less often overweight than first generation women, whereas among men, the second generation was more often overweight than the first generation. When adding the potential explanatory factors to this model, we found that the socioeconomic factors seem to weaken the association between generation and overweight in women. However, for men, none of the factors explained the generational differences, probably because none of the potential determinants were associated with overweight in men.

**Table 3:** Associations (Odds Ratios with 95%Confidence Intervals) between generational status and overweight for Turkish and Moroccan men and women (aged 15-30 years).

	Men N= 334 OR (95%CI)	Women N = 339 OR (95%CI)
Crude Model *		
First generation	1.00	1.00
Second generation	1.89 (1.09-3.24)	0.53 (0.19-0.90)
**Model 1**		
Second generation	1.90 (1.10-3.28)	0.56 (0.32-0.97)
**Model 2**		
Second generation	2.29 (1.19-4.42)	0.77 (0.40-1.46)
**Model 3**		
Second generation	1.92 (1.08-3.40)	0.59 (0.34-1.01)
**Model 4**		
Second generation	2.03 (1.15-3.60)	0.50 (0.28-0.90)

All models are adjusted for age and ethnicity.

* Crude model: adjusted for age and ethnicity

Model 1: crude model additionally adjusted for socio-demographic factors (marital status and children)

Model 2: crude model additionally adjusted for socioeconomic position (education level, position on the labour market and occupational status)

Model 3: crude model additionally adjusted for migration related factors (region of origin, reason for migration)

Model 4: crude model additionally adjusted for acculturation and religion (cultural orientation and social contacts, perceived importance of religion).

## Discussion

In this study we explored which social and cultural factors might underlie the differences in overweight between first and second generation Turkish and Moroccan people (age 15-30) in the Netherlands. In women, overweight appears to be lower among the second generation compared to the first generation. Differences in socioeconomic factors seem to partly account for this difference in overweight. However, the pattern found in men was reversed as overweight appears to be lower in first generation men. None of the factors studied could account for the higher prevalence found in second generation men.

We did not find exactly similar studies, but the reversed pattern in overweight for young men and women across generations was found before [7]. Most other studies in other ethnic minority populations, however, did not include first and second generation within this relatively young age group or they did not conduct separate analyses for men and women - which makes it difficult to compare the results [2,4,8,28]. One study did confirm that overweight in young male Turkish adolescents is higher than in young female Turkish girls, but no difference was made between first and second generation [29].

For the women in our study, their level of education seemed in particular to explain the differences in overweight between the generations, whereas the men of the second generation seem to have no health benefits as far as higher educational levels is concerned. Therefore, it seems that a higher education level in men does not necessarily imply a healthier lifestyle. The lack of a beneficial effect associated with educational level could be considered a cause of the higher rate of overweight. Further research is necessary to evaluate whether postulated explanations as to why a higher education in male immigrants does not 'protect' against overweight are relevant.

The finding that socioeconomic factors were associated with overweight in women and not in men is, however, in line with results from studies among the general population [12]. Also, in developing countries the association found between socioeconomic factors and overweight may differ for men and women. We found reports from a study conducted in Turkey, that the social patterning of overweight is most visible in women, with women of higher socioeconomic status having the lowest rates of overweight/obesity [30]. This pattern is similar to that observed in Western countries. However, recent evidence from Morocco indicates that a higher income is associated with more overweight in men but not in women [31]. In Brazil, boys with a higher socioeconomic status were more often overweight than boys with a low socioeconomic status. This association was the opposite in girls [32]. The authors explain this by the pressure from society placed in particular on young women and adolescents to be thin. In particular, parents who are more highly educated might be more aware of and monitor the lifestyle of their daughters so that the stigmatization of obesity is avoided [33]. It is possible that this explanation could also apply to the different patterns in overweight found for Turkish and Moroccan men and women in the Netherlands.

Due to a lack of associations between the acculturation indicators and overweight in both men and women, we did not find indications that a process of acculturation underlies the generational differences in overweight. This is in line with the findings from another Dutch study among (older) Turkish and Moroccan people in which acculturation was also not associated with overweight [8]. With regard to physical activity, however, there is evidence that a stronger orientation towards the dominant culture does lead to increased physical activity [34]. With regard to diet, mixed results have been found in previous studies. One Dutch study found that having more social contacts with ethnic Dutch people was associated with some healthy changes in diet among Turkish and Moroccan women, but it was not associated with an overall better quality of diet [35]. It has also been assumed that acculturation might lead to a less healthy diet as a result of the greater availability of snacks and fast food within Western society. Combining these findings, acculturation seems to have both positive and negative influences on weight among ethnic minority populations, implying that it does not lead to a healthier lifestyle per se.

Finally, the migration and religious factors were not associated with overweight and could therefore not account for the generational differences. We assume that generational status could also reflect other phenomena related to the development of overweight that were not included in our study.

Alternatively, it has been suggested that biological as well as social processes may be relevant to the changes that take place with improvements in living conditions [36]. This applies to either migration from less to more economically developed environments (such as from Turkey or Morocco to the Netherlands) or more generally with economic development. Specifically, it has been suggested that the life long effects of better living conditions during growth may make women less prone to adiposity and men more so, via environmentally driven changes to pubertal development [36]. Second generation ethnic minorities grew up in the Netherlands, whereas first generation migrants spent there first years in a different socioeconomic and cultural context [37,38]. These socioeconomic conditions across lifetime may be more predictive for obesity in adulthood than a person's current socio-economic position [39].

The current study has some limitations. Firstly, we used cross-sectional data which implies that no causal inferences could be made about the social determinants of overweight that were included in the study. For example, the association between socioeconomic position and overweight might be the opposite to what we originally assumed in this paper. Being obese could have a negative effect on opportunities in education and employment and as a result lead to a less favourable socioeconomic position among overweight people [40]. However, this pathway appears to be less common as results of longitudinal studies have indicated that changes in socioeconomic position have consequences for body weight and not vice versa [41].

Secondly, due to logistical problems during data collection, data on weight and height were not always measured by a trained interviewer but in one-third of the cases self-reported. This may have led to an underestimation of weight in this group [42]. However, there was no significant difference between the measured and the self-reported group in the prevalence of overweight either in men or women. The average BMI was higher in women who were measured by a professional, but the number of measured versus self-reported participants was equal according to the variables used in this study. Therefore, it is unlikely that the above has affected our results.

Finally, when interpreting the results it should be kept in mind that the first generation consisted of people who migrated to the Netherlands at different ages and who therefore have a different migration background and a varying number of years in the Netherlands. However, additional analyses showed that people who migrated before the age of 6 years did not differ from those older than 6 years regarding prevalence of overweight. Both groups differed significantly from second generation young people with regard to overweight, which implies that our first generation participants differed from second generation regardless of their age at migration in this particular group.

## Conclusions

In this study we explored potential factors underlying generational differences in overweight between first and second generation Turkish and Moroccan men and women aged 15-30 years. In second generation women, the lower prevalence of overweight could be partly accounted for by their better socioeconomic position. This result seems to indicate the importance of improving the socioeconomic position of women belonging to ethnic minority groups in order to reduce overweight. In second generation men, however, no explanations could be found for the higher prevalence of overweight. Further research is necessary to evaluate whether postulated explanations contribute to a better understanding of the generational patterning and, in particular, why a higher socio-economic position of men may not 'protect' against overweight.

## Competing interests

The authors declare that they have no competing interests.

## Authors' contributions

KS conceived the study, participated in its design, and commented on all drafts. KH conducted the study, participated in its design, performed the statistical analysis and wrote the manuscript. MN, VN and IV commented on all drafts of the manuscript. All authors read and approved the final manuscript.

## Pre-publication history

The pre-publication history for this paper can be accessed here:

http://www.biomedcentral.com/1471-2458/11/105/prepub
